# Look Out! Under $45,000 Bonds

**Published:** 1899-11

**Authors:** 


### Look Out! Under $45,000 Bonds.

On the thirtieth day of September eight dentists of Boston were sued by the International Tooth Crown Co. for sums varying from five to seven thousand dollars. Real estate was attached and marshals were placed in offices. These facts were telegraphed to Dr. Crouse, who at once secured able Boston counsel, and by a bond of $45,000, furnished by American Surety Co., secured release of the attachments and removal of keepers. This was followed by the arrival of Mr. Offield, who has been attorney of the Protective Association for ten years, to personally take charge of the cases, relieving us of all trouble and expense. The matter now rests pending the action of the courts. Imagine your predicament in such a case, if you were not a member of the Association. We feel that we should ask each non-member whether he can afford to take his chances unaided any longer, as no one can tell upon whom the blow will next fall. Did you read the circular sent out five weeks ago by the Association? If not, we recommend you to do so at once.

The Association will defend all its members. Non-members will *not* be defended. It is a duty you owe yourself to join the Protective Association at once. The doors will be closed December 1st.

JOHN F. DOWSLEY,
F. M. HEMENWAY,
THOMAS FILLEBROWN,
WALDO E. BOARDMAN,
L. D. SHEPARD,
*Committee.*

Boston, Mass., Nov. 11, 1899.

---

The information given above is of interest to every dentist in this country, and especially to those who are not yet under the shielding wing of the Dental Protective Association.

According to the above the opportunity for obtaining membership in that organization will have passed with December 1st, 1899. A very brief time, surely, but time enough to get within the refuge. Remember, the gate closes in ten days; then the outsiders must remain outside, and, when the flood comes, paddle their own canoes. The flood is well-nigh sure to come, and trouble awaits him who is not under cover. Come in.

